# Maternal infections during pregnancy and child cognitive outcomes

**DOI:** 10.1186/s12884-022-05188-8

**Published:** 2022-11-17

**Authors:** Janell Kwok, Hildigunnur Anna Hall, Aja Louise Murray, Michael Vincent Lombardo, Bonnie Auyeung

**Affiliations:** 1 Department of Psychology, University of Edinburgh, 7 George Square, EH8 9JZ Edinburgh, UK; 2 Laboratory for Autism and Neurodevelopmental Disorders, Centre for Neuroscience and Cognitive Systems, Italian Institute of Technology, Genoa, Italy; 3 Autism Research Centre, Department of Psychiatry, University of Cambridge, Cambridge, UK

**Keywords:** Pregnancy, Infections, Cognition, Child development, ALSPAC

## Abstract

**Background:**

Maternal prenatal infections have been linked to children’s neurodevelopment and cognitive outcomes. It remains unclear, however, whether infections occurring during specific vulnerable gestational periods can affect children’s cognitive outcomes. The study aimed to examine maternal infections in each trimester of pregnancy and associations with children’s developmental and intelligence quotients. The ALSPAC birth cohort was used to investigate associations between maternal infections in pregnancy and child cognitive outcomes.

**Methods:**

Infection data from mothers and cognition data from children were included with the final study sample size comprising 7,410 mother-child participants. Regression analysis was used to examine links between maternal infections occurring at each trimester of pregnancy and children’s cognition at 18 months, 4 years, and 8 years.

**Results:**

Infections in the third trimester were significantly associated with decreased verbal IQ at age 4 (*p* < .05, adjusted *R*^2^ = 0.004); decreased verbal IQ (*p* < .01, adjusted *R*^2^ = 0.001), performance IQ (*p* < .01, adjusted *R*^2^ = 0.0008), and total IQ at age 8 (*p* < .01, adjusted *R*^2^ = 0.001).

**Conclusion:**

Results suggest that maternal infections in the third trimester could have a latent effect on cognitive development, only emerging when cognitive load increases over time, though magnitude of effect appears to be small. Performance IQ may be more vulnerable to trimester-specific exposure to maternal infection as compared to verbal IQ. Future research could include examining potential mediating mechanisms on childhood cognition, such as possible moderating effects of early childhood environmental factors, and if effects persist in future cognitive outcomes.

## Background

Research has suggested links between a mother’s immune system during pregnancy and fetal growth and brain development [1, 2]. Central nervous system infections in pregnancy, such as herpes simplex virus and rubella, have been associated with intrauterine growth restriction, prematurity, and long-term neurodevelopmental deficits or disability [3]. Maternal prenatal bacterial infections have been linked with lower mean IQ scores at age 7 [4], and viral infections have shown associations with decreased IQ scores at age 7 with strong effect sizes [5] and learning disabilities [6]. In addition to reported effects on children’s cognition [7] and socioemotional difficulties [8], maternal infections have also been linked to a variety of psychiatric conditions such as bipolar disorder and schizophrenia [9, 10]. These studies demonstrate associations between maternal infections and general difficulties in cognition, behavior, and psychopathology.

The occurrence of viral maternal infection is proposed to influence the pathogenesis of infant central nervous system abnormalities via placental transfer-mediated vertical transmission [11]. This transmission may further disrupt the fetus’ organ and brain tissue development, negatively affecting the infant’s immune system, resulting in further pathology of a compromised immune system and increased risk of childhood inflammation [2]. Childhood low-grade inflammation then activates a systemic immune response associated with negative effects on brain development and function, with research showing small to moderate effect sizes on general intelligence [12]. These processes reflect multiple potential mechanisms of harm acting upon the child when maternal infection is present during pregnancy.

Even though trimester-specific associations have been reported for maternal infections and fetal neurodevelopment [5], evidence is still lacking on whether cognitive outcomes depend on the specific timing of maternal infection exposure during gestation. The idea of fetal neurodevelopment being stage-dependent is based on the fact that brain myelination only begins during the second trimester of pregnancy with further growth reinforcements in the third trimester, followed by postnatal growth [10, 13]. Therefore, inflammation such as infections during specific gestation periods may differentially affect the fetal growth process. However, only a limited number of studies have found that maternal inflammation during later gestational periods negatively affects a child’s cognitive-developmental infrastructure [14] and functional connections such as working memory [15].

Current research shows converging evidence between animal and human studies, with a review of large cohort studies suggesting a causal relationship between prenatal maternal inflammation and child outcomes [16], particularly with effects on the fetal brain development [17]. Examining the links between prenatal infection at different stages of gestation and cognitive outcomes is, however, subject to significant methodological challenges. In particular, it is not possible to conduct randomized experiments in humans for obvious ethical reasons. This makes it difficult to attribute causality to a factor such as prenatal infection. On the other hand, animal studies in which experimental manipulations are possible have been criticized for potential problems with generalization to humans [18]. As such, the field must rely on triangulation across these different evidence sources with complementary strengths and weaknesses [19–21].

Despite the fact that human studies must rely on observational evidence, they can provide valuable predictive information about which children may be most at risk of poorer cognitive outcomes. For example, identifying an association between prenatal infection at a particular stage of pregnancy and poorer cognitive ability can help identify children who may benefit from a higher level of monitoring and intervention early in life, such as screening for learning difficulties or prioritization for early-life preventive interventions. This study seeks to utilize observed evidence to provide predictive evidence of mother-fetal associations, despite limitations in examining specific underlying processes or mechanisms.

The aim of this study is to use a cohort sample to investigate whether the occurrence of maternal infections in each trimester of pregnancy is associated with children’s verbal, performance, and total IQ scores.

## Methods

### Setting

Data for this project were from the Avon Longitudinal Study of Parents and Children (ALSPAC), a population-based cohort study based in Avon, United Kingdom (UK), focusing on pregnant women with expected dates of delivery 1st April 1991 to 31st December 1992 [15, 22]. Regular follow-up clinic assessments were conducted over the years to track development over time. The ALSPAC dataset is a large, nationally representative sample of parents and children from the UK. Detailed medical information were gathered from pregnant women and their children from 8 weeks of gestation up to 22 years of age. Participants in ALSPAC have a slightly higher sociodemographic profile on average as compared to the general population in Avon and Great Britain [23]. For example, ALSPAC mothers are more likely to be married, live in owner-occupied accommodation, have a car in their household, and less likely to be non-White.

### Participants

The final sample of data collected and used in this study was for when the children were eight years old was *n* = 15,645. For the purposes of this study, participants with missing infection data across all trimesters (*n* = 2,125) were excluded from the sample, as well as those who had missing outcome data across all cognitive domains (*n* = 6,110). The final sample size comprised *n* = 7,410 mother-child participants.

### Outcome measures

#### Prenatal infections

Data on prenatal infections from the first, second and third trimesters were gathered in weeks 18 and 32 of gestation, and 8 weeks post-partum [23]. Retrospective assessment was completed at 8 weeks after birth for infections occurring in the third trimester of pregnancy. Women were asked whether they experienced any of the following infections: urinary tract infection, influenza, rubella, thrush, genital herpes, or other infections. Response options provided at 18 weeks were: ‘Yes, in 1st to 3 months’, ‘Yes, 4 months to now’, ‘Yes for both time periods’ and ‘Not at all’. Response options at week 32 and 8 weeks postpartum were: ‘Yes’, ‘No’, and ‘Don’t Know’. Response options for infections reported occurring during the first, second, or third trimester of pregnancy were coded and termed as ‘any trimester’. Infection data were coded into dichotomous variables, where any incidence of infection was coded as a ‘yes’, and no incidence was coded as a ‘no’. Infections were further coded according to trimesters, with any occurrence of infection coded as present (e.g., rubella or urinary tract infection at 18 weeks = infection present in the first trimester).

### Childhood IQ scores

Developmental and cognitive measures included the Griffiths Mental Development Scales (GMDS), administered at 18 months, the Wechsler Preschool and Primary Scale of Intelligence – Revised UK edition (WPPSI-RUK), administered at 4 years, and the Wechsler Intelligence Scale for Children, 3rd edition (WISC-III), administered at 8 years. The GMDS evaluates early development and consists of five subscales (locomotion, personal/social skills, hearing and speech, hand-eye coordination, and performance) [24]. The hearing and speech scale (language, response, speech development) [25] and the performance scale (skill development and manipulation), comprising 86 items each, were selected for use in the current study. Standardised developmental quotients were calculated through this formula: Developmental Quotient (DQ) = (Mental age x 100) / Chronological age. A new average GMDS development score variable was then calculated using the mean scores across all 5 scales by the ALSPAC team. All variables were age-adjusted by the ALSPAC research team. The study further included two measures of cognitive functioning: WPPSI-RUK for children aged 3–7 years [26] and a short-form version of the WISC-III for children aged 6–17 years [27, 28]. Both tests comprise two scales: verbal and performance (non-verbal ability and executive function), with each scale containing five subtests. Scores on both the WPPSI-RUK and the WISC-III range from 40 to 160. All IQ scores were used as continuous variables. Descriptive statistics for verbal IQ, performance IQ, and fullscale IQ scores are provided in Table 1.

### Confounders and covariates

Data on other perinatal and social factors which may be associated with prenatal infections and/or children’s cognitive outcomes were gathered from assessments from mothers and children. Child factors included child sex (male/female), gestational age (weeks) and birthweight (grams). Standard cutoffs for prematurity were applied to gestational age (≥ 37 weeks/<37 weeks) and birthweight (≥ 2500 g/<2500 g) as per clinical standards of falling within the 10th percentile of pregnancies, with increased infant mortality risk [29, 30]. Maternal and socioeconomic factors included maternal age at birth, maternal education, maternal history of smoking, maternal psychiatric history, and deprivation indices. Maternal education was assessed at 32 weeks of gestation through interviews and categorized as A-levels or lower. Maternal smoking was coded as ‘yes' or ‘no' and assessed through questionnaires for every trimester of pregnancy (18 weeks, 32 weeks, 8 weeks postpartum for the third trimester). Maternal psychiatric history was assessed in the first trimester, where women were asked if they had any psychiatric conditions (anorexia, bulimia, severe depression, schizophrenia, other psychiatric issues), and coded as yes if they indicated having any psychiatric illness. Deprivation indices were sorted into quintile ranks and measured by the official Indices of Multiple Deprivation, an area-level deprivation index measured in England accounting for several domains of deprivation: income, employment, health, education, housing, and living environment [31].

Previous literature has identified these factors to be associated with children’s cognitive development [32–34]. These factors were classified into potential confounders and covariates for the regression models in this study, with confounders being selected based on whether previous literature showed associations between infection exposure and cognitive outcomes (such as deprivation), and covariates (such as child’s sex) selected based on their potential association with children’s cognitive outcomes. Adjusting for the latter was of interest to determine the unique contribution of infection exposure over and above other established influences.

### Statistical analysis

All statistical analyses were conducted using R (version 3.4.1) [35]. Data was inspected for and met parametric statistical assumptions. Linear regression models were used to examine the relations between infections occurring at each trimester and children’s cognitive scores at ages 18 months, 4 years, and 8 years. Three types of models were fit: an unadjusted model, a model adjusted for confounders, and a model adjusted for confounders and additional covariates. These models were run in a hierarchical manner, with increasingly adjusted models specified by adding variables to models from previous steps. The fully adjusted model included possible confounders of maternal and socioeconomic factors, and the additional covariates of child sex, gestational age, birthweight, maternal psychiatric history, and maternal smoking (Table 2). As the models were trimester-specific, regression analyses for infections in each trimester were also adjusted for infections in the other two trimesters.

In the primary analysis, missing data were handled with full information maximum likelihood estimator, and an alpha level of 0.05 indicated statistical significance (*p* < .05). Sensitivity analysis included multiple imputation models using the MICE package in R, with 25 imputations applied in order to avoid Monte Carlo error [36, 37]. Models were fit from the multiple imputed data sets, with estimates pooled into a single set of estimate and standard errors, which was then used for the analysis.

## Results

**Table 1:** Descriptive statistics for predictors and outcomes

	N	%	
**Maternal Prenatal Infections**			
** Any time during pregnancy (** ***n *** **= 6,849)**			
No	3107	45.4	
Yes	3742	54.6	
** 1st trimester (** ***n *** **= 6,889)**			
No	5288	76.8	
Yes	1601	23.2	
** 2nd trimester (*n*** **= 6,860)**			
No	4468	65.1	
Yes	2392	34.9	
**3rd trimester (*n* = 7,091)**			
No	5588	78.8	
Yes	1503	21.2	
**Child scores**	**Mean**	**SD**	**Range**
**GMDS (Mean age = 1.5 years)**			
Hearing /Speech (Verbal)	100	16.1	62–114
Performance	113	12.7	64–135
Average	108	8.8	65–114
**WPPSI-RUK (Mean age = 4.1 years)**			
Verbal Scale	100	13.6	54–152
Performance Scale	108	14.6	55–151
Fullscale	104	14.2	52–113
** WISC-III (Mean age = 8.6 years)**			
Verbal Scale	107	16.7	46–155
Performance Scale	100	17.1	46–151
Total Scale	104	16.5	45–151

**Table 2:** Confounders and covariates (*n* = 7,410)

**Confounders**		
	**Mean (SD)**	**Range**
**Maternal age at childbirth (years)**	29.1 (1.23)	15–44
	**N**	**%**
**Maternal education**		
(≥ A’ levels)	3068	41.3
(< A’ levels)	4111	55.5
Missing	231	3.2
**Deprivation index**		
Highest quintile	1965	26.5
60–80%	1162	15.7
40–60%	1054	14.2
20–40%	885	11.9
Lowest quintile	765	10.3
Missing	1579	21.3
**Covariates**		
**Child Gender**		
Male	3740	50.4
Female	3670	49.6
**Child Gestation**		
≥ 37 weeks	6982	94.2
< 37 weeks	428	5.8
**Child Birthweight**		
≥ 2500 g	6970	94.0
< 2500 g	349	4.7
Missing	91	1.3
**Maternal psychiatric history**		
No	5955	80.4
Yes	829	11.2
Missing	626	8.4
**Maternal smoking (any point in pregnancy)**		
No	5073	68.5
Yes	1488	20.1
Missing	849	11.4

**Table 3:** Hierarchical regression models on trimester-based infections in pregnancy and cognitive outcomes (*n* = 7,410)

			Any trimester	1st trimester	2nd trimester	3rd trimester
			**B values (** ***SE*** **)**			
**Model 1** ^a^	**GMDS**	Hearing & Speech (verbal)	− 1.55 (.*985*)	− 0.429 (*1.20*)	− 1.32 (*1.15*)	− 0.940 (*1.31*)
		Performance	− 0.099 (.*778*)	0.029 (.*943*)	− 0.191 (.*901*)	− 0.177 (*1.03*)
		Average	− 0.854 (.*543*)	− 0.647 (.*655*)	− 0.623 (.*626*)	− 0.297 (.*717*)
	**WPPSI-RUK**	Verbal	− 1.45 (.*887*)	− 0.547 (*1.07*)	− 0.051 (*1.02*)	− 2.51 (*1.16*) *
		Performance	− 0.438 (.*951*)	0.647 (*1.15*)	0.430 (*1.10*)	− 0.810 (*1.25*)
		Fullscale	− 1.07 (.*923*)	0.037 (*1.11*)	− 0.061 (*1.07*)	− 1.95 (*1.21*)
	**WISC-III**	Verbal	− 0.287 (.*418*)	0.945 (.*509*)	− 0.120 (.*482*)	− 1.48 (.*562*) **
		Performance	− 0.378 (.*427*)	0.449 (.*517*)	− 0.062 (.*491*)	− 1.49 (.*571*) **
		Total	− 0.368 (.*412*)	0.796 (.*501*)	− 0.116 (.*475*)	− 1.64 (.*553*) **
**Model 2** ^b^	**GMDS**	Hearing & Speech (verbal)	− 0.150 (.*148*)	− 1.09 (*1.38*)	− 1.54 (*1.33*)	− 0.915 (*1.55*)
		Performance	− 0.228 (.*885*)	− 0.169 (*1.06*)	0.078 (*1.02*)	− 0.606 (*1.19*)
		Average	− 0.637 (.*605*)	− 0.913 (.*729*)	− 0.313 (.*706*)	− 0.530 (.*820*)
	**WPPSI-RUK**	Verbal	− 1.37 (.*960*)	− 1.14 (*1.15*)	− 1.42 (*1.10*)	− 1.75 (*1.26*)
		Performance	− 0.508 (*1.04*)	0.163 (*1.25*)	− 0.116 (*1.20*)	− 0.490 (*1.38*)
		Fullscale	− 1.04 (.*993*)	− 0.614 (*1.19*)	− 0.871 (*1.14*)	− 1.30 (*1.31*)
	**WISC-III**	Verbal	− 0.063 (.*431*)	0.317 (.*523*)	− 0.262 (.*498*)	− 0.859 (.*584*)
		Performance	− 0.192 (.*457*)	0.151 (.*552*)	0.228 (.*524*)	− 1.65 (.*615*) **
		Total	− 0.139 (.*425*)	0.289 (.*515*)	− 0.070 (.*489*)	− 1.34 (.*574*) *
**Model 3** ^c^	**GMDS**	Hearing & Speech (verbal)	− 1.71 (*1.22*)	− 1.21 (*1.44*)	− 0.349 (*1.39*)	− 1.61 (*1.64*)
		Performance	− 0.132 (.*952*)	− 0.077 (*1.11*)	0.396 (*1.08*)	− 0.315 (*1.27*)
		Average	− 0.612 (.*642*)	− 0.791 (.*756*)	0.286 (.*734*)	− 0.565 (.*862*)
	**WPPSI-RUK**	Verbal	− 1.08 (*1.05*)	0.852 (*1.22*)	− 0.983 (*1.17*)	− 1.64 (*1.36*)
		Performance	− 0.167 (*1.13*)	0.682 (*1.32*)	− 0.028 (*1.27*)	− 0.448 (*1.47*)
		Fullscale	− 0.692 (*1.08*)	− 0.150 (*1.26*)	− 0.540 (*1.21*)	− 1.24 (*1.40*)
	**WISC-III**	Verbal	0.304 (.*467*)	0.415 (.*557*)	− 0.036 (.*531*)	− 0.603 (.*623*)
		Performance	0.172 (.*493*)	0.259 (.*587*)	0.496 (.*559*)	− 1.41 (.*655*) *
		Total	0.255 (.*459*)	0.412 (.*548*)	0.188 (.*522*)	− 1.34 (.*574*)

Linear regression analyses and beta coefficients for trimester-based infections in pregnancy and cognitive outcomes

*p<.05, **p<.01, ***p<.001

^a^ Unadjusted models

^b^ Adjusted for potential confounds of maternal education, maternal age, deprivation, infections in other trimesters

^c^ Adjusted for confounds, and additional covariates of maternal smoking, maternal psychiatric history, gestation, birth weight, and child sex

Descriptive statistics showed that the occurrence of maternal prenatal infections at any time during pregnancy was 54.6%, with most infections occurring during the 2nd trimester (34.9%) (Table 1). Mean developmental quotient and intelligence quotient scores were similar to the general population (GMDS = 108, WPPSI-RUK = 104, WISC-III = 104) (Table 1). Table 2 shows further descriptive statistics for maternal variables and children’s birth outcomes.

Unadjusted models found no links between infections occurring in the first or second trimesters and children’s outcomes. Significant associations were found between third trimester infections and WPPSI-RUK verbal scores (β= − 0.076, *p* = .031) at age 4, and WISC-III verbal IQ scores (β= − 0.036, *p* = .008), performance IQ scores (β= − 0.036, *p* = .009), and total IQ scores (β= − 0.040, *p* = .003) at age 8 (Table 3). These results showed that an infection during the third trimester of pregnancy was associated with a 2.5 IQ point decrease for WPPSI-RUK verbal scores at age 4, 1.5 IQ point decrease in WISC-III verbal scores, 1.5 IQ point decrease for WISC-III performance scores, and 1.6 IQ point decrease for WISC-III total scores at age 8. No significant associations were found between prenatal infections in all three trimesters and any of the children’s cognitive outcomes (Model 1).

Significant associations were attenuated after the first adjustment for confounders maternal age, maternal education, deprivation, and infections in the other two trimesters (Model 2). Associations were no longer found for WPPSI-RUK verbal IQ scores (β= − 0.053, *p* = .166) at 4 years and WISC-III verbal IQ scores (β= − 0.021, *p* = .141) at 8 years. Significant associations remained between third-trimester infections and WISC-III performance IQ scores (β= − 0.039, *p* = .007) and WISC-III total IQ scores (β= − 0.033, *p* = .020) at 8 years (Table 3). These results showed that an infection during the third trimester of pregnancy was associated with a 1.7 IQ point decrease in WISC-III performance scores, 1.3 IQ point decrease for WISC-III total scores at age 8.

Fully adjusted models (Model 3) with confounders (maternal age, maternal education, deprivation indices, other two trimesters) and additional covariates (maternal smoking, maternal psychiatric history, gestation, birth weight, and child sex) showed that infections in the third trimester were associated with lower WISC-III performance scores at 8 years (β= − 0.033, *p* = .032) (Table 3). These results represent a 1.4 IQ point decrease in WISC-III performance IQ scores.

### Sensitivity analysis

A sensitivity analysis of the study models found consistent associations between infections occurring at the third trimester and outcomes for WISC-III verbal IQ scores (β= -1.33, *p* = .022), performance IQ scores (β= -1.19, *p* = .028), and total IQ scores (β= -1.39, *p* = .009) at age 8 in the unadjusted model. While no significant associations were shown between occurrence of infections at any trimester and cognitive outcome scores for the partially and fully adjusted models, lower maternal education (*p* < .05) and highest deprivation (*p* < .05) were generally seen to have associations with lowered cognitive scores across all trimesters.

## Discussion

A prospective birth cohort was used to examine associations between maternal infections occurring at each trimester of pregnancy and children’s developmental and intelligence scores at 18 months, 4 years, and age 8. Results from unadjusted models provided evidence for maternal infections during the third trimester being associated with verbal IQ scores at age 4, and verbal, performance, and total IQ scores at age 8. The associations between third-trimester infections and cognitive outcomes were seen to be attenuated after adjustment for confounders, with significant associations seen only for performance and total IQ scores at age 8 after the first adjustment. Further adjustment with additional covariates left only significant associations for performance IQ scores at age 8. A similar prevalence of maternal infections during pregnancy was shown in this study as compared to other cohort samples [3, 18].

Children’s cognitive outcomes appear to depend on critical periods in gestation, with evidence pointing to effects of infection limited to later gestation. Animal models have provided evidence on potential mechanisms, showing that inflammatory cytokines produced in response to infection may be linked to fetal brain development changes. Mice treated with analogues of bacterial or viral infections at different gestational periods showed different neurodevelopmental profiles of hippocampal reelin and GAD67 cell number expression in the hippocampal dorsal or ventral stratum oriens of offspring [18]. Studies investigating infection-associated immunological events on the fetus showed early maternal inflammation to be linked with negative effects on the development of a fetus’ dopaminergic system at multiple levels such as cell distribution and connectivity [38]. In contrast, later brain insults during pregnancy have been linked to deficits in cell organization and maturation of synapses which occur over an extended period, affecting cognitive function [14].

While animal models provide a possible explanation as to how brain neurochemistry and structure can potentially be affected by different times of infection exposure, evidence from human studies, especially looking at specific trimester effects and cognitive outcomes, is lacking. Further, while some genetic patterns have been found in maternal intellectual ability and child IQ scores [39], no firm conclusions can be drawn that suggests genetics fully accounts for children’s cognitive abilities. Instead, there is current growing emphasis on using the gene-environment interaction to examine the role of maternal inflammation and biological pathways that lead to fetal brain development and cognition [40, 41].

Literature on human studies has been mixed, showing varying results on vulnerability to inflammation occurring in different gestational periods, especially on a child’s cognitive development [10, 13, 15]. The significant associations between third trimester infection and cognitive outcomes at age 8 identified in this study suggest a possible latent effect of maternal systemic inflammation which may possibly influence cognitive development. This could be due to an interaction with unaccounted environmental factors after birth, such as lowered socioeconomic status, which could confound child development as it is also considered an inflammatory process during childhood [42]. The link with maternal infections during later gestation shows that despite having fetal brain infrastructure in place, environmental factors such as deprivation could still affect brain structure or functional connections after birth.

This suggests that infections in late pregnancy may influence the postnatal processes of cognitive development. With differences shown in results using the developmental (GMDS) and intelligence quotients (WPPSI-RUK, WISC-III), it could be possible that effects of prenatal maternal infection are not evident in early development, with no identification of potential developmental delay as reflected in GMDS. However, results from formal IQ assessments such as the WPPSI-RUK and WISC-III could possibly be showing effects that emerge only under increased cognitive load, with cognitive IQ assessments created to assess advanced skills such as abstract reasoning or working memory.

While this study could not directly infer causality, study results are still consistent with previous research on maternal-fetal associations, where clinical evidence shows how presence of maternal inflammation during pregnancy using elevated cytokine profiles contribute to prenatal risk programming [43, 44]. Other studies using these same maternal cytokines found negative influences on prenatal central nervous system development [45], with animal models showing further evidence for negative neurobehavioral outcomes [46]. In sum, research supports how prenatal programming consists of multiple variables such as occurrence and regulation of inflammation, genetic susceptibility, and environmental stressors; all of which interact in a complex manner that is still not yet fully understood in humans [16], which could explain why study results were mixed. Nevertheless, this study contributes further evidence through the specific scope of how maternal infections during different trimesters of pregnancy could possibly affect childhood developmental and intelligence quotient outcomes.

### Strengths and limitations

Main study strengths were the use of a large high-quality longitudinal design and a well-characterized sample. While cohort studies such as the ALSPAC tend to have considerable attrition due to reasons such as loss to follow-up related to socioeconomic status, it has been shown that differences in estimates when comparing full or restricted cohorts tended to be small [47]. Similar infection rates during pregnancy and study attrition allowed for comparability with other cohort studies also looking at mother-child associations. Study limitations include a reliance on mother self-reported infection data; that the records of infections were not classified by severity; and that causes of infections (e.g. bacterial/viral/fungal) were not recorded and could not be stratified by pathogen type for analysis. In addition, while the study has included a list of confounders and covariates, not all factors such as parental attitudes, styles or other variables related to the quality of care of the child, or genetic factors could be accounted for in this study. In addition, this version of GMDS was created to identify developmental delays and therefore should be interpreted with caution as to whether it is a true assessment of cognition at 18 months. Despite not being able to account for these above factors, study results still showed predictive associations for the cognitive measures assessing IQ, which was consistent with research from both animal and human studies focusing on maternal-fetal interactions. Finally, assessments from the dataset were only measured up to age 8, so lasting effects of cognitive development could not be analyzed beyond those years.

## Conclusion

Our study highlighted possible third-trimester specific effects of maternal infection on children’s cognitive abilities. However, effect sizes were small, suggesting a weak link between maternal infections during the third trimester of pregnancy and childhood cognitive outcomes. One potential direction of future research could include examining mechanisms which mediate these effects of prenatal infections on childhood cognition such as fetal brain gene expression after maternal immune activation [48]. Other research could also explore temporal dependency and specificity to performance IQ scores from 4 years onwards, whether the effect of infection is moderated by specific early childhood environmental factors, and if its effect persists in future cognitive outcomes.

## Data Availability

Data sharing is not available upon request due to prior approval required from the ALSPAC team. No additional data are available.

## Abbreviations

ALSPAC: Avon Longitudinal Study of Parents and Children
DQ: Development Quotient
IQ: Intelligence Quotient
GMDS: Griffiths Mental Development Scales
WPPSI-RUK: Wechsler Preschool and Primary Scale of Intelligence – Revised UK edition
WISC-III: Wechsler Intelligence Scale for Children, 3rd edition
